# The deubiquitinase USP8 regulates ovarian cancer cell response to cisplatin by suppressing apoptosis

**DOI:** 10.3389/fcell.2022.1055067

**Published:** 2022-12-12

**Authors:** Cristina Corno, Padraig D’Arcy, Marina Bagnoli, Biagio Paolini, Matteo Costantino, Nives Carenini, Elisabetta Corna, Paola Alberti, Delia Mezzanzanica, Diego Colombo, Stig Linder, Noemi Arrighetti, Paola Perego

**Affiliations:** ^1^ Department of Experimental Oncology, Unit of Molecular Pharmacology, Milan, Italy; ^2^ Department of Biomedical and Clinical Sciences, Linköping University, Linköping, Sweden; ^3^ Department of Experimental Oncology, Unit of Molecular Therapies, Milan, Italy; ^4^ Pathology Unit 1, Fondazione IRCCS Istituto Nazionale Dei Tumori, Milan, Italy; ^5^ Department of Medical Biotechnology and Translational Medicine, University of Milano, Milan, Italy; ^6^ Department of Oncology-Pathology, Karolinska Institutet, Stockholm, Sweden

**Keywords:** ovarian cancer, ubiquitin-specific protease 8, cisplatin, drug resistance, apoptosis

## Abstract

The identification of therapeutic approaches to improve response to platinum-based therapies is an urgent need for ovarian carcinoma. Deubiquitinases are a large family of ubiquitin proteases implicated in a variety of cellular functions and may contribute to tumor aggressive features through regulation of processes such as proliferation and cell death. Among the subfamily of ubiquitin-specific peptidases, USP8 appears to be involved in modulation of cancer cell survival by still poorly understood mechanisms. Thus, we used ovarian carcinoma cells of different histotypes, including cisplatin-resistant variants with increased survival features to evaluate the efficacy of molecular targeting of USP8 as a strategy to overcome drug resistance/modulate cisplatin response. We performed biochemical analysis of USP8 activity in pairs of cisplatin-sensitive and -resistant cells and found increased USP8 activity in resistant cells. Silencing of USP8 resulted in decreased activation of receptor tyrosine kinases and increased sensitivity to cisplatin in IGROV-1/Pt1 resistant cells as shown by colony forming assay. Increased cisplatin sensitivity was associated with enhanced cisplatin-induced caspase 3/7 activation and apoptosis, a phenotype also observed in cisplatin sensitive cells. Increased apoptosis was linked to FLIP_L_ decrease and cisplatin induction of caspase 3 in IGROV-1/Pt1 cells, cisplatin-induced claspin and survivin down-regulation in IGROV-1 cells, thereby showing a decrease of anti-apoptotic proteins. Immunohistochemical staining on 65 clinical specimens from advanced stage ovarian carcinoma indicated that 40% of tumors were USP8 positive suggesting that USP8 is an independent prognostic factor for adverse outcome when considering progression free survival as a clinical end-point. Taken together, our results support that USP8 may be of diagnostic value and may provide a therapeutic target to improve the efficacy of platinum-based therapy in ovarian carcinoma.

## Introduction

Ovarian cancer is the most deadly gynecological cancer (Siegel et al., 2021). Diagnosis commonly occurs at advanced disease stages, a feature that results in a poor prognosis (Romero and Bast, 2012; Bowtell et al., 2015). Ovarian cancer comprises epithelial, germ cell, and stromal cell tumors, with epithelial ovarian cancers, i.e., carcinomas representing the majority. Ovarian carcinoma is a heterogeneous disease including various histological subtypes that can be grouped as Type I and Type II tumors, the latter comprising high grade disease (Kroeger and Drapkin, 2017). Platinum-based therapy remains the standard approach in ovarian carcinoma (Rottenberg et al., 2021). Since drug resistance often develops, efforts are still needed to define new strategies to optimize treatment (Herzog and Monk, 2017). In this regard, the inhibition of Epidermal Growth Factor Receptor (EGF-R) *per se* has not provided clinically useful results (Mehner et al., 2017), an observation suggesting that a better understanding of the biological network maintaining tumor cell survival involving Receptor Tyrosine Kinases (RTKs) might unravel new targets for modulation of chemotherapy efficacy.

Deubiquitinating enzymes (DUBs) comprise around 100 enzymes (Clague et al., 2019). Three of the known DUBs are associated to the proteasome. Following inhibition of such DUBs poly-ubiquitinated proteins accumulate with cell toxicity (D'Arcy et al., 2011). On the other hand, ubiquitin (Ub) is removed from proteins involved in cell survival, DNA damage repair and apoptosis by various non-proteasome-associated DUBs (D'Arcy et al., 2011). Regulation of tumor cell survival by DUBs occurs through several mechanisms including interplay with receptors (Row et al., 2006; Meijer and van Leeuwen, 2011; Sirisaengtaksin et al., 2017). Internalization of cell surface receptors and subsequent degradation in lysosomes can be triggered by mono-, multi- and poly-ubiquitination (Haglund et al., 2003). Among DUBs, Ubiquitin-Specific Protease 8 (USP8, UBPY) removes K48- and K63-linked polyubiquitin chains participating in refilling of the cellular Ub pool (Meijer and van Leeuwen, 2011). Conflicting reports have been published about the outcome of EGF-R regulation by USP8 (Cao et al., 2007; Panner et al., 2010; Byun et al., 2013), most of them supporting that this DUB promotes receptor degradation. However, poor down-regulation or over-expression of RTKs may result in inefficient termination of receptor signaling, leading to enhanced oncogenic stimuli. In this context, a report by Byun S et al. suggests USP8 as a novel therapeutic target for overcoming gefitinib resistance in lung cancer, specifically involving USP8 in EGF-R signaling (Byun et al., 2013). Besides, a synergistic interaction between cisplatin and caffeic acid phenethyl ester, capable to inhibit USP8, has been observed in endometrioid ovarian carcinoma cells (Colombo et al., 2022).

Selected DUBs have been reported to be associated with poor prognosis in different cancer types. For instance, Ubiquitin-Specific Protease 1 (USP1) over-expression has been found in stomach adenocarcinoma samples as compared to normal tissue, with higher USP1 expression being associated with shorter overall survival (Meng and Li, 2022). High expression of Ubiquitin-Specific Protease 7 (USP7), a frequent event in hepatocellular carcinoma, has been linked to a poor overall survival (Wang et al., 2018). In glioma, where several DUBs have been found to contribute to tumor progression by gene expression profiling interactive analysis, a prognostic role for Ubiquitin-Specific Protease 28 (USP28) was proposed (Liang et al., 2022).

Since survival pathways activated by RTKs have been shown to be frequently de-regulated in cancer cells, particularly in platinum drug-resistant cells (Cossa et al., 2014; Corno et al., 2017), the aim of this study was to investigate the possible contribution of USP8 to drug resistance of ovarian carcinoma cells with particular reference to platinum-resistant preclinical models, using a RNA interference-based approach. In addition, since USP8 seems to exert a non-redundant physiological role (Dufner and Knobeloch, 2019), the effects of USP8 molecular targeting were also examined in drug-sensitive cells.

## Materials and methods

### Cell lines, growth conditions and cell sensitivity assays

The following human ovarian carcinoma cell lines were used in this study: IGROV-1, A2780, and the cisplatin-resistant variants IGROV-1/Pt1, A2780/CP, A2780/BBR (Perego et al., 1996; Perego et al., 2003; Cossa et al., 2014; Corno et al., 2017); PEO1, PEO4 and PEO6 cell lines derived from a high-grade serous ovarian cancer patient before and after development of clinical platinum resistance (Stronach et al., 2011), (Sigma-Aldrich,Milan, Italy). RPMI-1640 medium (Lonza, Basel, Switzerland), supplemented with 10% FBS (Gibco, Life Technologies, Carlsbad, California) was used for cell culture, after thawing from frozen stocks. Culture was for no more than 20 passages. Colony forming assays according to guidelines (Perego et al., 2018) as described (Corno et al., 2017) were used to evaluate drug sensitivity. Briefly, 24 h after seeding in 6-well plates (500 cells/well), cisplatin (Teva Italia S. r. l.) at different concentrations was added to the culture medium and cells were incubated for 10 days. After alcohol fixation and staining with 2% crystal violet, colonies of at least 30 cells were observed under the microscope, and counted. IC_50_ values represent the drug concentrations producing 50% cell survival decrease.

### Analysis of USP8 activity

For the analysis of basal USP8 activity, exponentially growing cells were seeded in 75 cm^2^ flasks and 24 h later harvested, washed with PBS and lysed by adding 200 μl of lysis buffer consisting of 50 mM Tris pH 7.5, 50 mM NaCl, 5 mM MgCl2, 1% TritonX-100, 10% glycerol, and freshly prepared 2 mM ATP and 1 mM DTT. Samples were lysed on ice for 20 min and then centrifuged for 10 min (14,000 rpm, 4 °C). Supernatants were collected and an aliquot was used for assaying protein concentration using the BCA assay (Thermo Fisher, Waltham, Massachusetts, United States). Twenty μg of protein lysate was added to reaction buffer (50 mM Tris pH 7.5, 50 mM NaCl, 5 mM MgCl_2_) containing freshly prepared 2 mM ATP and 1 mM DTT. For each sample, HA-Ub-VS (2 µM final concentration, Enzo Life Science, Euroclone, Milan, Italy) was added and samples were incubated for 30 min at 37°C and then fractionated by SDS-PAGE (3–8% polyacrylamide) and transferred to membranes. A polyclonal antibody to USP8 (1:1000, Bethyl Laboratories, cat. N. A302-929A, Aurogene, Rome, Italy) was used to detect the labeled and unlabeled USP8 by western blotting.

### USP8 knockdown

Small interfering RNAs (siRNAs) directed against USP8 [Silencer_ Select s17372 to exons 14–17 (siRNA b); s105116 targeting exons 7–9 (siRNA c), Thermo Fisher Scientific] or negative control siRNA (Silencer Select Negative Control #2 siRNA, Thermo Fisher Scientific) were used. Twenty-four h after plating in 6-well plates (25.000 cells/cm^2^), transfection with 10 nM siRNAs was carried out in OptiMEM transfection medium (Gibco by Life Technologies) using Lipofectamine RNAiMAX (Thermo Fisher Scientific, Monza, Italy). Cells transfected with the negative control siRNA undergo the same procedure of the USP8 targeting siRNA transfected cells except that the negative control siRNA does not have a transcript to target. Transfection was for 5 h and after that time complete medium was used to replace the transfection medium. Target gene levels were evaluated by quantitative Real time PCR (qRT-PCR) 48 and 144 h after transfection start in experiments to assess the effect of drug treatment. Forty-eight h after transfection start, cells were harvested and reseeded to perform 1) cell sensitivity assays with cisplatin by colony forming assays in 6-well plates (500 cells/well); 2) for Annexin V-binding assays in 6-well plates; 3) for caspase 3/7 and caspase 8 activation assays in 96-well plates; 4) or processed for Antibody Arrays.

### Quantitative real time PCR

The RNeasy Plus Mini Kit (Qiagen, Hilden, Germany) and High Capacity cDNA Reverse Transcription Kit (Applied Biosystems, Foster City, California, United States) were employed for RNA isolation and reverse transcription, respectively. One μg of RNA, in the presence of RNAse inhibitors, was used according to manufacturer’s protocol. Reactions of technical triplicate samples occurred in a 10 μl volume with 2.5 µl cDNA, 5 µl master mix (TaqMan universal Fast PCR Master Mix, Thermo Fisher Scientific), 0.5 μl of the specific TaqMan assay [USP8: Hs. PT.58.4573260; SNAIL1: Hs00195591_m1; SNAIL2: Hs00161904_m1; NANOG: Hs02387400_g1; SOX2: Hs01053049_s1; POU5F1: Hs01895061_u1; TWIST: Hs00361186_m1, Thermo Fisher Scientific]. The apparatus for reactions was a 7900HT Fast Real-Time PCR System (Thermo Fisher Scientific) equipped with SDS (Sequence Detection Systems) 2.4 software and the RQ manager software. The determination of relative levels of cDNA was carried out using the relative quantification (RQ) method with the comparative Ct (ΔΔCt) assay configuration. After identification of a cycle threshold value (Ct, i.e., the PCR cycle at which each probe fluorescence exceeds the detection threshold), ΔCt, ΔΔCt and RQ were calculated: ΔCt = Ct gene - Ct housekeeping ΔΔCt = ΔCt sample - ΔCt calibrator RQ = 2^−ΔΔCt^. GAPDH was used as housekeeping control gene. Untransfected cells were used as calibrators.

### Western blot analysis and antibodies

After harvesting with a scraper, cells were lysed. The lysis buffer consisted of 0.125 M Tris HCl pH 6.8 (Sigma-Aldrich), 5% sodium dodecyl sulfate (SDS, Lonza) and protease/phosphatase inhibitors (25 mM sodium fluoride, 10 μg/ml pepstatin A, 1 mM phenylmethylsulfonyl fluoride, 10 μg/ml trypsin inhibitor, 12.5 μg/ml leupeptin, 30 μg/ml aprotinin, 1 mM sodium orthovanadate and 1 mM sodium molybdate, all from Sigma-Aldrich). After boiling for 5 min, samples were sonicated for 25 s; quantification was carried out with the BCA method (Pierce, Thermo Fisher Scientific). Proteins were fractionated by SDS-PAGE and blotted on nitrocellulose membranes. After blocking in PBS with 5% (w/v) dried non-fat milk, blots were incubated overnight at 4°C with primary antibodies. Immunoreactive bands were revealed by enhanced chemiluminescence detection system ECL (GE Healthcare, United Kingdom). The quantification of band intensities was performed after scanning the films using the ImageJ 1.47v software. The following antibodies were used: anti-actin, anti-FLIP and anti-vinculin from Sigma-Aldrich, anti-USP8 from Bethyl Laboratories, anti-Akt from BD Biosciences (New Jersey, United States), anti-phospho Akt (Ser 473), anti-phospho EGFR (Ser 1046/1047), anti-Bcl-2, anti-claspin, anti-cleaved caspase 3 from Cell Signaling (Danvers, Massachusetts, United States), anti-survivin from Abcam (Cambridge, United Kingdom). Secondary antibodies were from GE Healthcare.

### Apoptosis analyses

Apoptosis was evaluated by Annexin V-binding assay (Annexin V-FITC kit, Immunostep, Salamanca, Spain) 24 h after seeding in untransfected cells, and cells transfected with siRNA or negative control oligonucleotides, treated for 72 h with cisplatin. After washing with cold PBS and resuspension in binding buffer (10 mM Hepes-NaOH, pH 7.4, 2.5 mM CaCl_2_, and 140 mM NaCl, Immunostep), 10^5^ cells were incubated in binding buffer at room temperature in the dark for 15 min with 5 µl of FITC-conjugated Annexin V and 10 μl propidium iodide (Immunostep). Flow cytometry (BD Accuri, Becton Dickinson) was used for Annexin V binding detection, by acquiring 10^4^ events/sample. Data were analyzed using the Cell- QuestPro software (Becton Dickinson). The activation of caspases was determined using luminescent Caspase Glo 3/7 and Caspase Glo 8 assays (Promega, Fitchburg, WI, United States). Cells were seeded (8 × 10^3^ cells/well) in 96-well plates and treated with cisplatin for 48 h. Caspase activation was detected according to manufacturer’s instructions.

### Antibody arrays

Cell lysates for Proteome Profiler Antibody Arrays were prepared following the manufacturer’s instructions (R&D System). For Western immunoblot analysis visualization Labscan imaging technology and software (GE Healthcare, Wauwatosa, Wisconsin) were used. Quantification of band intensities was carried out using ImageJ software by pixel-integrated intensity.

### Statistical analysis of preclinical data

GraphPad Prism (version 5.02, GraphPad Software Inc., La Jolla, CA, United States) was employed for statistical analyses of data from *in vitro* experiments as detailed throughout the manuscript.

### Immunohistochemistry

Immunohistochemistry (IHC) on formalin-fixed, paraffin-embedded (FFPE) sections was used to analyze USP8 levels of epithelial ovarian carcinoma (EOC) specimens. Briefly, sections underwent xylene deparaffinization and alcohol rehydration. Antigen retrieval was in 10 mM, pH 6.0, citrate buffer at 96°C for 15 min in autoclave. Slides were incubated with 3% H_2_O_2_ for 20 min to quench endogenous peroxidase. After washing and saturation in saturating solution (PBS 1% BSA, 30 min) at room temperature (RT), samples were incubated for 1 h at RT with primary rabbit polyclonal anti-USP8 (Bethyl Laboratories, cat. N. A302-929A) at a 1 : 200 dilution. A biotinylated anti-rabbit secondary antibody (30 min, 1: 200, Dako S. p.A, Milan, Italy) followed by HPR streptavidin (1:300, Dako) for 30 min at RT was then used. Following development of the peroxidase reaction with 3, 3′-diaminobenzidine (Dako), section counterstaing with hematoxylin was carried out. Slides incubated with secondary antibody alone provided negative controls. To set up the IHC protocol the IGROV-1 cell line, which expresses USP8 as observed through Western blotting, was used. Staining was recorded by a semiquantitative grading system in which samples were defined as positive when 10% of cells displayed reactivity. Intensity was scored as 1, 2 or 3. Two independent observers blinded to patient characteristics and outcome evaluated the slides and discussed all cases with discrepant evaluations during observation with a double-headed microscope. A consensus was reached.

### Patients characteristics and statistical analysis

An institutional EOC case material with retrospectively collected samples was used. Consent for the use of clinical information for clinical-translational research had been obtained from all patients and the Institutional Review Board (IRB) approved this study. Patients’ clinical and pathological characteristics at diagnosis were reported for each case for which FFPE blocks were available. Patients were grouped based on similar clinical and pathological characteristics (Table 1). For analyzing the distribution of USP8 positive and negative patients in relation to clinical and pathological variables, the Chi-square test was used. *p*-values <0.05 were considered significant. Grouping of patients for survival analysis patients was based on similar clinical and pathological characteristics. Tumor staging and grading were in conformity with the International Federation of Gynecology and Obstetrics (FIGO) and WHO criteria, respectively. Based on the extent of residual disease after primary surgery, patients were classified in two categories: Optimal Debulking (OD) that includes patients with no evident residual disease or residual tumor smaller than 1 cm, and Sub-Optimal Debulking (SOD) that includes patients with residual tumor larger than 1 cm. Histotype and surgical debulking were coded as dichotomous indicator variables [serous histotype vs. other histotypes; sub-optimal debulking (SOD) vs. optimal debulking (OD)]. Regarding USP8, patients classification was according IHC results. The time interval (months) from the date of surgery and the date of progression or death, whichever occurred first or the date of last follow-up for patients alive without progression was meant as Progression free survival (PFS). Median follow-up time was 54 months (39–104), median PFS 17 months (95% CI: 15–20); altogether, 53 events of PFS were observed. The Kaplan-Meier method was used to report PFS curves that were compared using the log-rank test. Median estimates, with 95% confidence interval (CI), are shown. To estimate the hazard ratio (HR) for each relevant prognostic variable a Cox univariate model was used. To evaluate the prognostic impact in the presence of known clinical prognostic factor (residual disease and histotype), multivariable analysis was carried out using a Cox regression model. All analysis were performed using R statistical language version 3.2.2 (URL http://www.R-project.org).

**TABLE 1:** Classification of patients based on clinical and pathological characteristics and distribution of USP8 expression ^a^.

Clinical and pathological characteristics			USP8 IHC						

			Negative (*n* = 36)		Positive (*n* = 25)		NA (*n* = 4)		*Chi square test*
	Total (*n* = 65)	%	n	%	n	%	n	%	
**Stage**									*ns*
III	51	78	29	57	20	39	2	4	
IV	14	22	7	50	5	36	2	14	
NA	0	0	0	0	0	0	0	0	
**Histology**									*ns*
Serous	43	66	21	49	18	42	4	9	
Undifferentiated	3	5	2	67	1	33	0	0	
Clear Cells	3	5	1	33	2	67	0	0	
Endometroid	9	14	6	67	3	33	0	0	
Mucinous	0	0	0	0	0	0	0	0	
Others + Mixed	6	9	5	83	1	17	0	0	
NA	1	2	1	100	0	0	0	0	
**Grade**									*ns*
1 well differentiated	1	2	1	100	0	0	0	0	
2 moderately differentiated	14	22	8	57	5	36	1	7	
3 poorly differentiated	48	74	27	56	18	38	3	6	
NA	2	3	0	0	2	100	0	0	
**Residual disease**									*ns*
Optimal debulking	22	34	10	45	11	50	1	5	
Sub-Optimal debulking	43	66	26	60	14	33	3	7	
NA	0	0	0	0	0	0	0	0	

^a^ IHC, immunohistochemistry; NA, not assessable.

## Results

### USP8 is deregulated in ovarian carcinoma cell lines

**FIGURE 1:**
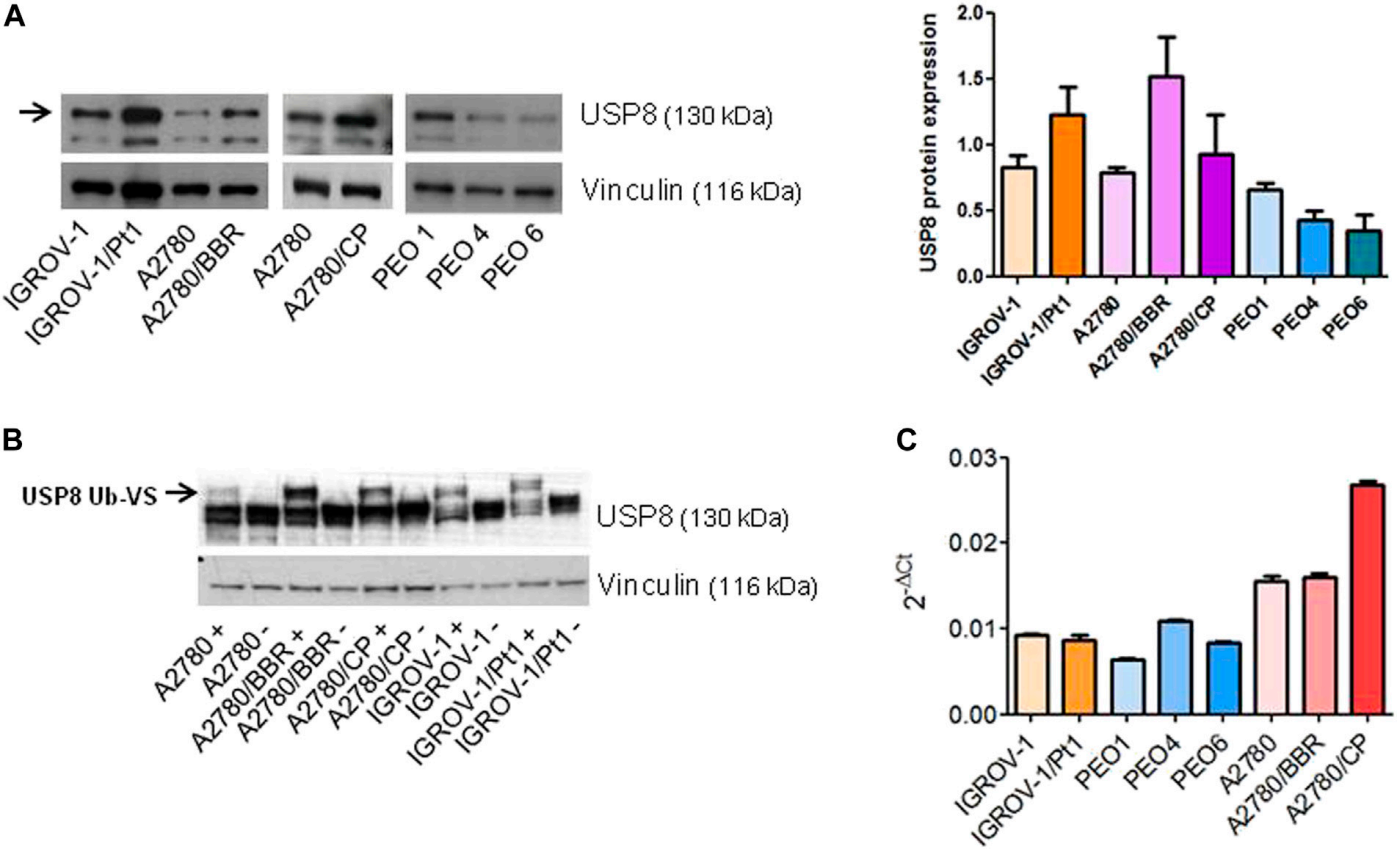
USP8 levels and activity in different ovarian carcinoma cell lines. **(A)** Western blot analysis of USP8 expression in different ovarian carcinoma cell lines, IGROV-1, A2780, PEO1 and the resistant variants. Vinculin was used as loading control. One experiment representative of at least three is reported. Band intensity was quantified using ImageJ and normalized to vinculin. The obtained values are reported in the histogram on the right. **(B)** USP8 activity in ovarian carcinoma cell lines, IGROV-1, A2780 and the resistant variants. Labeling of deubiquitinases from whole-cell lysates with HA-Ub-VS was followed by electrophoresis and immunoblotting with USP8. +, samples incubated with HA-Ub-VS; -, samples incubated without HA-Ub-VS. Control loading is shown by vinculin. The blot is overexposed to allow detection of the active band (indicated by the arrow). One experiment representative of at least three is reported. **(C)** Analysis of USP8 mRNA levels by qRT-PCR. GAPDH was used as housekeeping gene.

We assessed the levels and activity of the USP8 protein in different ovarian cancer cell lines including three cisplatin-resistant variants (IGROV-1/Pt1, A2780/CP and A2780/BBR), established by chronic exposure to platinum drugs, using western blot analysis. These cells are known to exhibit multiple molecular alterations associated with drug resistance including activation of survival pathways (Perego et al., 1996; Perego et al., 2003; Cossa et al., 2014; Corno et al., 2017). The most representative genetic differences among all the ovarian carcinoma cell lines and the most common molecular alterations are shown in Table 2. The cisplatin-resistant variants IGROV-1/Pt1, A2780/CP, selected for resistance to cisplatin and A2780/BBR selected for resistance to a trinuclear platinum complex (BBR3464) and exhibiting cross-resistance to cisplatin (Perego et al., 2003) were found to display increased levels of USP8 (Figure 1A). Conversely, sublines derived from a patient at the first and second recurrence (PEO4 and PEO6) (Stronach et al., 2011) displayed reduced USP8 levels as compared to cells obtained from the platinum-sensitive tumor (PEO1). To assess USP8 activity we used an active site directed probe, Ub-VS, which covalently links active DUB enzymes with Ub. Increased USP8 activity was observed in the cisplatin-resistant variants, as evidenced from the appearance of a high molecular weight band representing Ub-VS labelled USP8 in comparison with the respective parental cell line (Figure 1B). To examine a possible link between gene expression and USP8 levels, we carried out qRT-PCR analysis. Overall, no direct relation between mRNA and protein level was found (Figure 1C). Then, we assessed if USP8 enrichment in cisplatin-resistant models leads to a stem cell phenotype besides enhancement of epithelial-mesenchymal transition (EMT) genes. A qRT-PCR analysis of the expression of genes involved in pluripotency/markers of stemness (NANOG, SOX2, POU5F1 coding for OCT4) including EMT markers (Twist, Snail1, Snail2) in the parental cell lines and in the resistant variants characterized by USP8 enrichment indicated an increase of selected genes only in the A2780 resistant variants (Supplementary Figure S1), thereby suggesting that USP8 expression might be, but not always, associated with a stem cell phenotype.

**TABLE 2:** Characteristics of the cell lines and drug-resistant variants.

Cell line	p53 status	BRCA gene status	Apoptosis-related proteins	Thiols and nucleophic molecules	Other
IGROV-1	Wild-type^a^	BRCA1^**+/−**^ ^b^			Mutant PIK3CA^c^ Missense mut (*p*.R38C)^d^, non-stop mut (*p*.*1069W)
		BRCA2^+/+^ ^b^			
		BRCA2* missense mut (*p*.P3150T)^d^			
IGROV-1/Pt1	Mutant^a^	Not determined	Reduced Bax^a^	Increased GSH^e^	No mutations in KRAS gene codons 12/13, BRAF gene codon 600, full-length MAP2K1^f^
A2780	Wild-type^g^	BRCA1^**+/+**^ ^b^			Mutant PIK3CA^c^
		BRCA2^+/+^ ^b^			BRAF missense mut (*p*.V226M)^d^
A2780/BBR	Wild-type**^g^**	Not determined		Increased metallothioneins and human neurofilament low^g^	Not determined
A2780/CP	Wild-type^h^	Not determined	Increased Bcl-2^h^	Not determined	Not determined
PEO1		BRCA2 deleterious mutation compensated by reversion mutation^b^			No mutations in PIK3CA, BRAF, HRAS, NRAS, KRAS according to ^d^
PEO4		BRCA2 reversion mutation^b^	Bcl-2 family proteins^i^		No mutations in PIK3CA, BRAF, HRAS, NRAS, KRAS according to ^d^
PEO6		BRCA2 reversion mutation^b^			No mutations in PIK3CA, BRAF, HRAS, NRAS, KRAS according to ^d^

^a^ Perego et al., 1996.

^b^ Stordal et al., 2013.

^c^ Zorea et al., 2018.

^d^ https://depmap.org/portal/

^e^ Perego et al., 1998.

^f^ Cossa et al., 2014.

^g^ Perego et al., 2003.

^h^ Perego et al., 1997.

^i^ Dai et al., 2017.

### Molecular inhibition of USP8 in cisplatin-resistant cells

**FIGURE 2:**
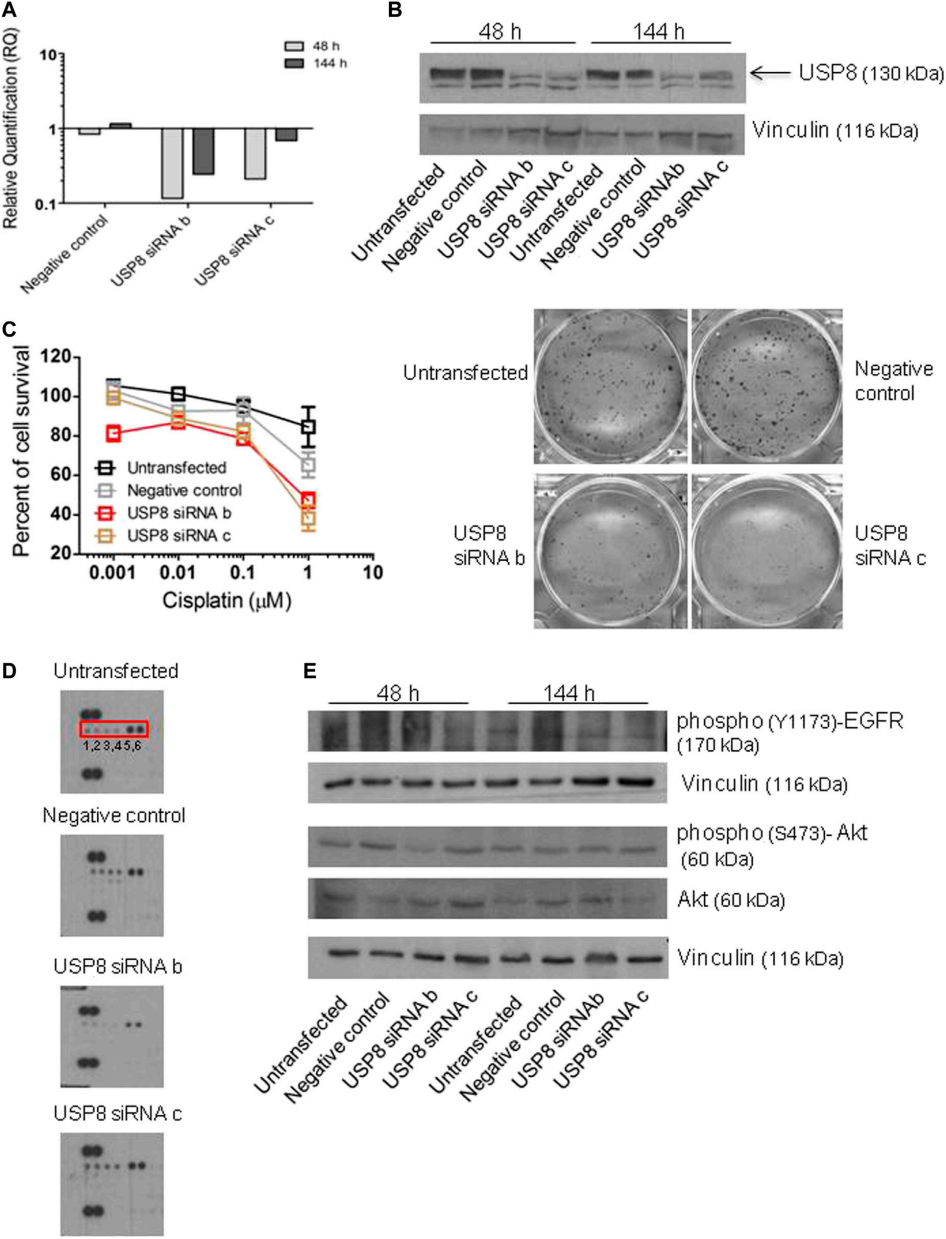
Survival-related features of IGROV-1/Pt1 cells upon USP8 molecular targeting. **(A)** Quantitative RT-PCR of USP8 mRNA levels in IGROV-1/Pt1 at different time points after siRNA transfection; untransfected cells were the calibrator; GAPDH was used as housekeeping gene. **(B)** USP8 protein levels were analyzed by western blot at different time points after siRNA transfection. The reported experiment is representative of three. Vinculin was used as loading control. Quantification of the band intensity was performed with ImageJ, with normalization to vinculin and the obtained values are reported in Supplementary Figure S2 **(C)** Colony forming ability assayed 48 h after transfection start. Twenty-4 h after seeding IGROV-1/Pt1 cells were treated with cisplatin (continuous exposure). IC_50_ is the concentration inhibiting cell growth by 50% (IC_50_ > 1 µM for untransfected or negative control cells, IC_50_ = 0.78 and 0.52 µM for USP8 siRNAb and siRNAc cells, respectively). (*p* < 0.0001 by One-Way ANOVA followed by Bonferroni’s test for multiple comparisons). The reported values are the mean ± SD of three replicates. The reported experiment is representative of two. Representative images of cells treated with 1 μM cisplatin are shown. **(D)** Human phospho-RTK Array analysis 48 h after transfection start. Phospho-EGFR = 1,2; phospho-ErbB2 = 3,4; phospho-ErbB3 = 5,6. Quantification of dot intensity was performed using ImageJ. The obtained values are reported in Supplementary Table S1 **(E)**. Western blot analysis of protein levels in USP8-silenced cells. Quantification of the band intensity was performed using ImageJ, with normalization to vinculin. The band intensities of phosphorylated Akt were referred to total forms. The obtained values are reported in the Supplementary Figure S2.

**FIGURE 3:**
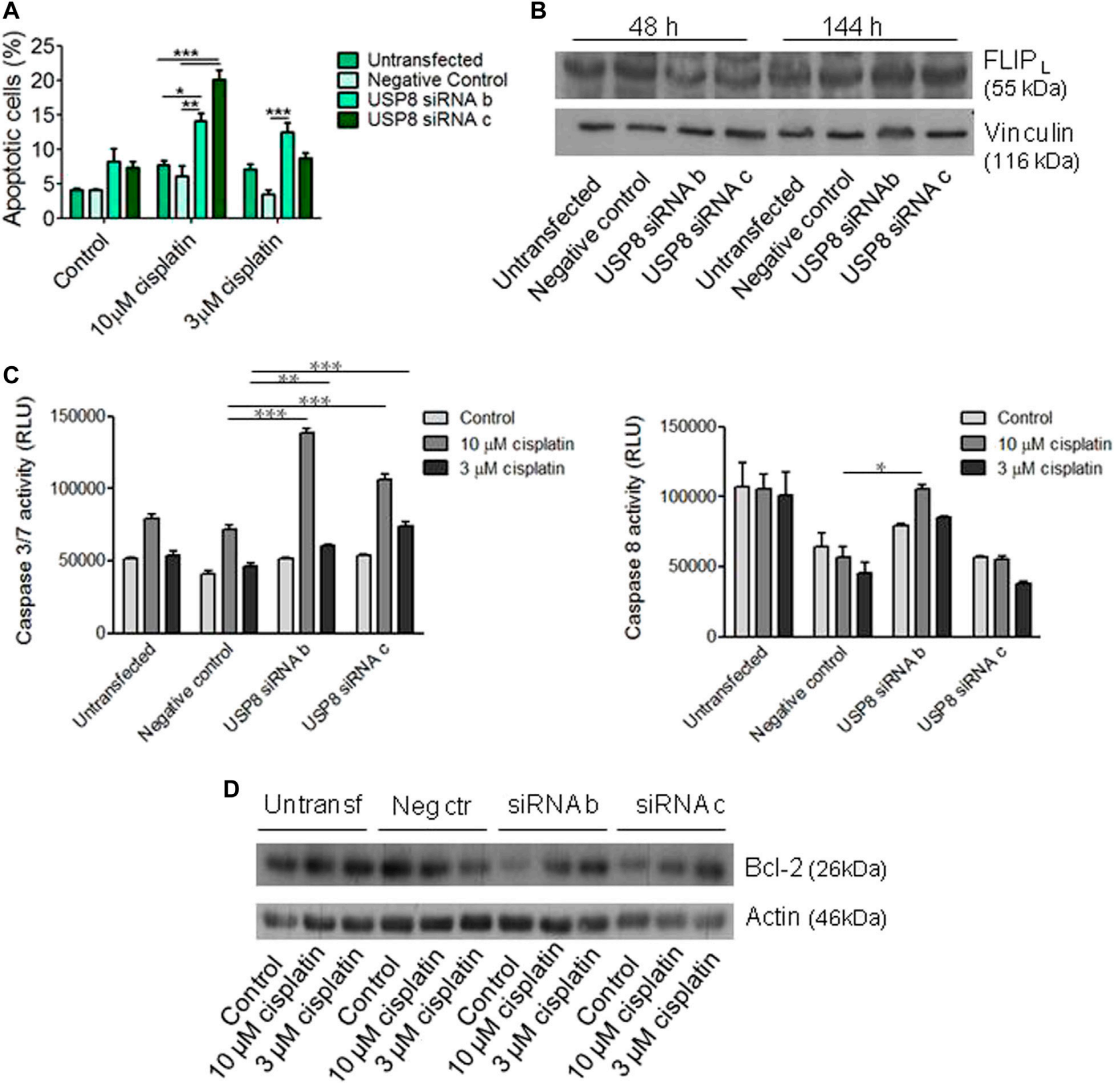
Analysis of apoptosis following USP8 molecular targeting in IGROV-1/Pt1 cells. **(A)** USP8-silenced cells were seeded for apoptosis analysis by Annexin-V binding assay 48 h after transfection start and treated for 72 h with cisplatin. The columns indicate late apoptosis (Annexin V-PI positive cells). One experiment representative of at least two is shown. **(B)** Western blot analysis of FLIP_L_ protein levels in USP8-silenced cells. The band intensity was quantified using ImageJ and normalized to vinculin. The obtained values are reported in the Supplementary Figure S2 **(C)** Forty-eight hours after transfection, cells were seeded for caspase 3/7 and caspase eight activation analysis by luminescent Caspase Glo assays and treated for 48 h with cisplatin. One experiment representative of at least two is shown. *, *p* < 0.05; **, *p* < 0.01; ***, *p* < 0.001 by One-Way ANOVA followed by Bonferroni’s test for multiple comparisons. **(D)** Western blot analysis of Bcl-2 was carried out in USP8-silenced cells seeded 48 h after transfection start and treated for 48 h with cisplatin. The band intensity was quantified using ImageJ, normalized to actin and the obtained values are reported in the Supplementary Figure S2.

The effect of the molecular inhibition of USP8 was examined by knocking down USP8 expression in cisplatin-resistant IGROV-1/Pt1 cells with the goal of clarifying its contribution to cisplatin resistance (Figure 2-3). SiRNA duplexes targeting USP8 mRNA, transiently transfected in IGROV-1/Pt1 cells, markedly decreased the levels of mRNA 48 h after transfection start, with a persistent transcript down-regulation (Figure 2A). The efficiency of silencing was checked over time by qRT-PCR analysis starting from 48 h to 144 h from transfection start. Three siRNAs were originally tested but one (siRNA a) was excluded from further analyses due to poor effects. USP8 protein levels were also effectively reduced with silencing by siRNA b and c (Figure 2B), a phenomenon resulting in increased sensitivity to cisplatin as shown by changes in cell survival obtained with the colony forming assay (Figure 2C). In silenced cells, a decrease of phosphorylation of RTKs belonging to the EGF-R family reflecting reduced activation was observed, particularly with siRNA b using antibody arrays (Figure 2D, Supplementary Table S1). This suggested that sensitization to cisplatin may be the consequence of reduced stimulation of survival by RTK downstream signaling. A reduced activation of EGF-R and Akt upon silencing was observed by western blot analysis, particularly for siRNA b (Figure 2E), with 41% decrease as compared to negative control-transfected cells.

Drug-induced apoptosis was investigated following knocking down of USP8 in IGROV-1/Pt1 cells by Annexin V-binding assay (Figure 3). Increased apoptosis after exposure to cisplatin was observed in siRNA-silenced cells as compared to negative control siRNA-transfected cells exposed to 10 μM cisplatin (*p* < 0.01 by One-Way ANOVA followed by Bonferroni’s test for multiple comparisons) (Figure 3A). Because USP8 has been shown to stabilize the long form of FLICE-like inhibitory protein (FLIP_L_) (Jeong et al., 2017), we also examined FLIP_L_ levels in silenced cells. We observed a decrease of this anti-apoptotic protein consistently with increased cisplatin-induced apoptosis (Figure 3B). When examining activation of caspases by using luminescent Caspase Glo 3/7 and Caspase Glo 8 assays, we found enhanced caspase 3/7 activation in USP8-silenced IGROV-1/Pt1 cells as compared to cells transfected with the negative control siRNA exposed to 3 and 10 μM cisplatin (Figure 3C). In addition, increased caspase 8 activation was observed in USP8 siRNA b-transfected cells treated with 10 μM cisplatin compared to negative control siRNA-transfected cells exposed to the same cisplatin concentration. Western blot analysis of the anti-apoptotic protein Bcl-2 indicated a decrease at basal level upon silencing (with reference to untreated negative control-transfected cells set to 1, 0.3 for untreated siRNA b-transfected cells, 0.7 for untreated siRNA c transfected cells; Figure 3D).

### Molecular inhibition of USP8 in cisplatin-sensitive cells

**FIGURE 4:**
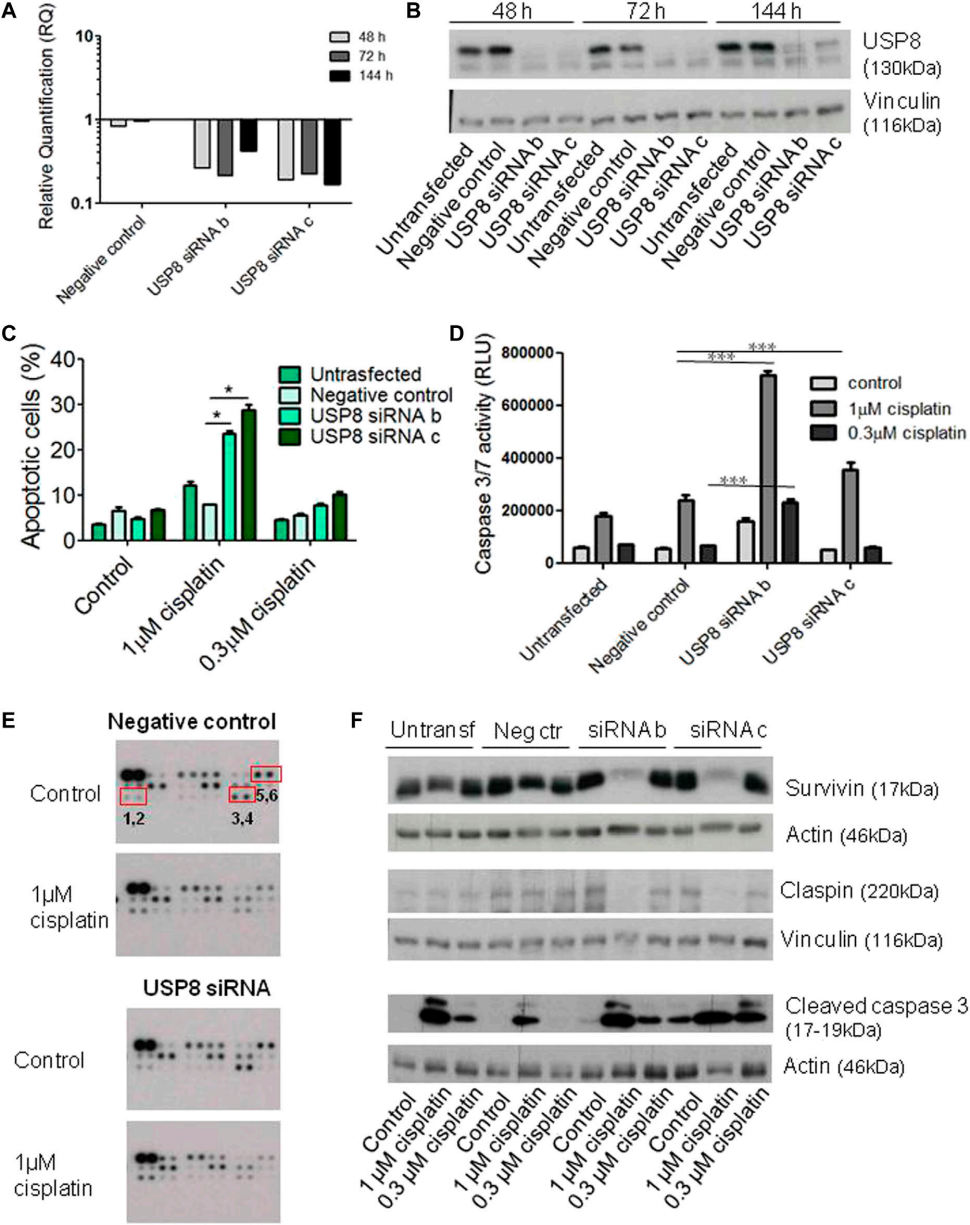
Molecular targeting of USP8 in IGROV-1 cells. **(A)** Quantitative RT-PCR of USP8 mRNA levels in IGROV-1 at different times after siRNA transfection; untransfected cells were used as calibrator and GAPDH as housekeeping control gene. **(B)** USP8 protein levels were analyzed by western blot at different times after siRNA transfection. One experiment representative of at least three is reported. Control loading is shown by vinculin. The band intensity was quantified using ImageJ, normalized to vinculin and the obtained values are reported in the Supplementary Figure S3 **(C)** USP8-silenced cells were seeded for apoptosis analysis by Annexin-V binding assay 48 h after transfection start and treated for 72 h with cisplatin. The columns indicate late apoptosis (Annexin V-PI posistive cells). One experiment representative of at least two is shown. **(D)** Twenty-four hours after transfection, cells were seeded for caspase 3/7 activation analysis by luminescent Caspase Glo asay and treated for 48 h with cisplatin. One experiment of at least two is shown. ***p < 0.0001 by One-Way ANOVA followed by Bonferroni’s test for multiple comparisons. **(E)** Human Apoptosis Array analysis was carried out in USP8-silenced cells with siRNA c seeded 48 h after transfection start and treated for 72 h with cisplatin. Phospho-p53 (S46) = 1,2; survivin = 3,4; claspin = 5,6. Dot intensity was quantified using ImageJ and the obtained values are reported in the Supplementary Table 2 **(F)** Validation of arrays by western blotting and cleaved caspase three analysis was carried out in USP8-silenced cells with siRNA b and c seeded 48 h after transfection start and treated for 72 h with cisplatin. Band intensity was quantified using ImageJ, normalized to actin/vinculin and the obtained values are reported in the Supplementary Figure S3.

**FIGURE 5:**
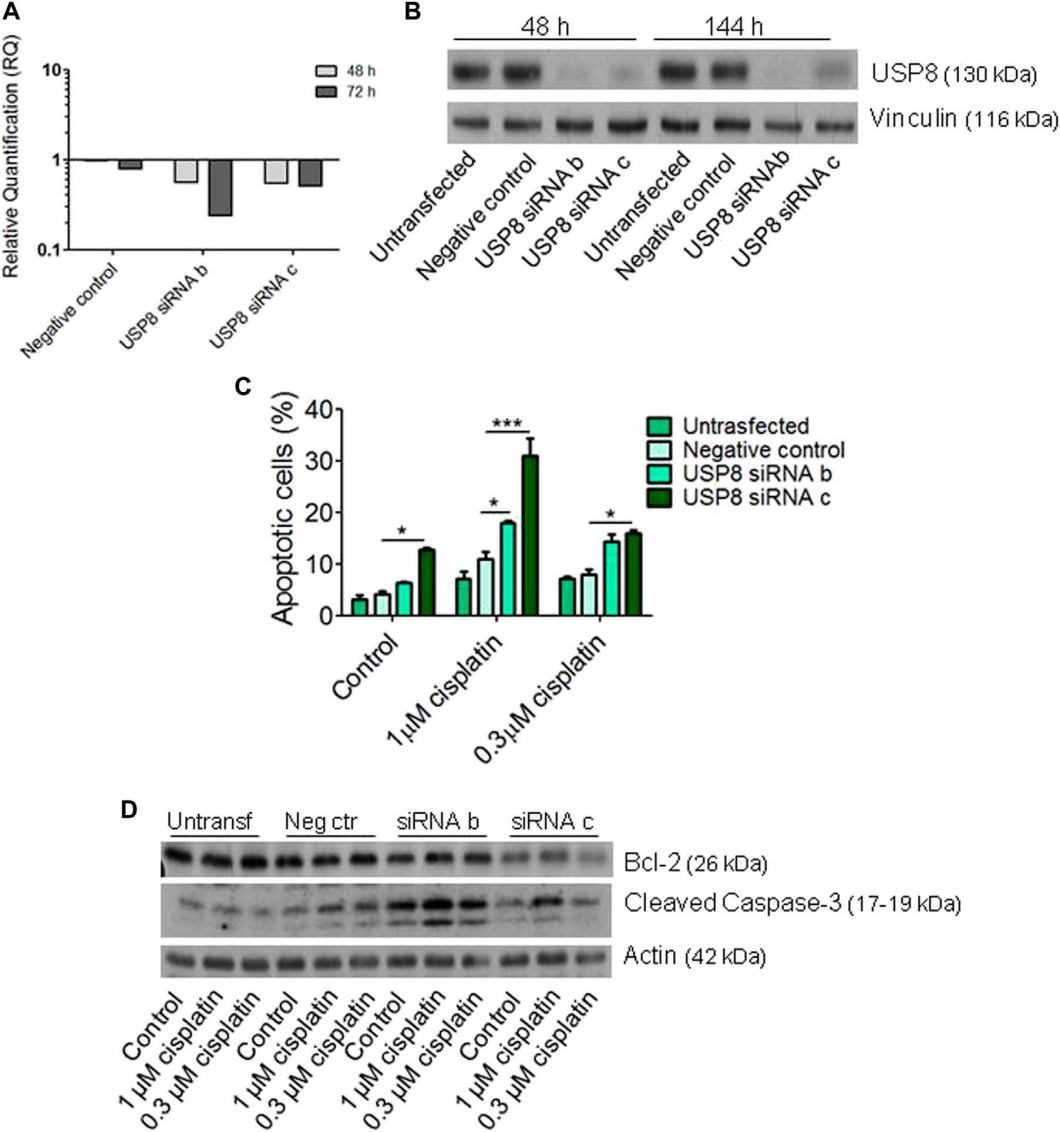
Molecular targeting of USP8 in PEO1 cells. **(A)** Quantitative RT-PCR of USP8 mRNA levels in PEO1 at different experimental time points after siRNA transfection; untransfected cells were the calibrator; GAPDH was used as housekeeping gene. **(B)** USP8 protein levels were analyzed by western blot at different experimental time points after siRNA transfection. The experiment is representative of three. Vinculin represents the loading control. Quantification of the band intensity was performed using ImageJ, with normalization to vinculin. The obtained values are reported in the Supplementary Figure S4 **(C)** Apoptosis analysis of USP8-silenced cells by Annexin-V binding assay 48 h after transfection start in cells treated for 72 h with cisplatin. The columns indicate late apoptosis (Annexin V-PI positive cells). The reported values are the mean ± SD of three technical replicates. **(D)** Bcl-2 and cleaved caspase 3 protein levels in USP8-silenced PEO1 cells. Forty-eight h after transfection start, cells were treated for 72 h with cisplatin and then lysed. The quantification of the band intensity was carried out using ImageJ, with normalization to actin. The obtained values are reported in the Supplementary Figure S4.

The effects of molecular targeting of USP8 were assessed in cisplatin-sensitive cell lines of different histotypes, i.e., the endometrioid IGROV-1 and the high grade serous PEO1 (Figure 4-5). Transient transfection of USP8 siRNAs produced a marked down-regulation of USP8 in IGROV-1 cells (Figures 4A, B). USP8 silenced IGROV-1 cells were more susceptible to cisplatin-induced apoptosis as measured by the Annexin V-binding assay following 72 h exposure to cisplatin (comparison of cells exposed to 1 μM cisplatin, *p* < 0.05 by One-way ANOVA followed by Bonferroni’s test; Figure 4C). Moreover, the activation of caspase 3/7, using luminescent Caspase-Glo 3/7 assay, was increased in USP8-silenced cells as compared to cells transfected with negative control siRNA exposed to 1 µM cisplatin (Figure 4D). Antibody array analysis suggested a modulation of survivin, claspin and phopho-p53 levels at a similar extent upon silencing (Figure 4E, Supplementary Table S2). A validation of the changes found with the arrays confirmed the slight down-regulation of survivin and claspin observed upon treatment with cisplatin of USP8-siRNA transfected cells (Figure 4F), whereas the up-modulation of phospho-p53 was not confirmed (data not shown). Cleaved caspase three displayed a slight increased upon silencing at the basal level (with reference to untreated negative control-transfected cells set to 1, 1.3 for untreated siRNA b-transfected cells, 4.2 for untreated siRNA c transfected cells).

The effects of molecular targeting of USP8 was also assessed in the high grade serous PEO1 cell line (Figure 5). In these cells, a better effect of silencing was observed at 48 h from transfection start for siRNA b than for siRNA c with a more marked down-regulation of USP8 mRNA levels. However, down-regulation of protein efficiently occurred for both siRNAs (Figures 5A, B). When cisplatin-induced apoptosis was evaluated after treatment with one or 0.3 μM cisplatin for 48 h, increased apoptosis was observed as compared to negative control transfected cells exposed to the same drug concentrations (*p* < 0.05 by One-Way ANOVA followed by Bonferroni’s test for multiple comparisons; Figure 5C). Silenced cells were also more prone to apoptosis under basal condition, i.e., in the absence of treatment. No marked changes of Bcl-2 levels were observed; cleaved caspase three tended to be higher in siRNA-transfected *versus* negative control-transfected cells (with reference to untreated negative control-transfected cells set to 1, 3.0 for untreated siRNA b-transfected cells, 1.5 for untreated siRNA c transfected cells).

### Expression of USP8 in ovarian carcinoma clinical specimens

**FIGURE 6:**
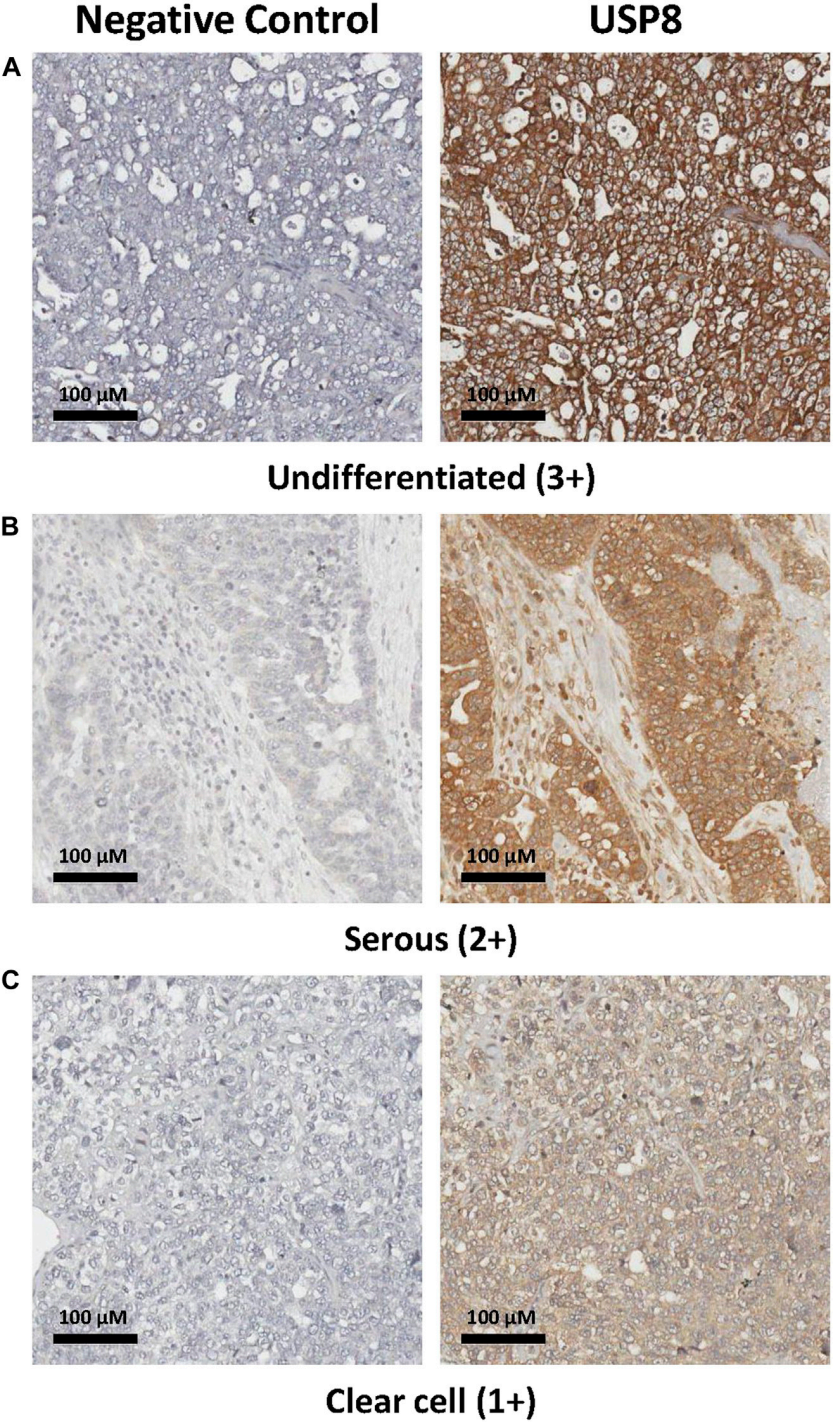
Immunohistochemical evaluation of USP8 in ovarian carcinoma specimens. **(A)** Representative image of an undifferentiated case with high intensity staining (3+), showing that the protein of interest was present both at the plasma membrane and in the cytoplasm. **(B)** Image of a serous ovarian carcinoma with medium intensity staining (2+); **(C)** Image of a clear cell carcinoma with low intensity staining (1+). All samples are from stage III patients. Images showing that the protein of interest was present both at the plasma membrane and in the cytoplasm are reported. Negative control, slides incubated with secondary antibody alone.

To explore the role of USP8 in the clinical setting in ovarian carcinoma, we carried out IHC staining on 65 clinical specimens from advanced stage ovarian carcinoma. We found that around 40% of tumors (25 out of 61 evaluable cases) were USP8 positive. A representative image, showing that the protein of interest was present both at the plasma membrane and in the cytoplasm, is reported in Figure 6. Patient characteristics and association of USP8 expression with clinical and pathological variables are shown in Table 1. Exploration of Kaplan Meier curves relative to PFS of patients stratified for USP8 expression did not reveal any significant difference between patients at high *versus* low USP8 expression with median PFS of 18 months (95%CI:15–22) and 16 months, respectively (95% CI: 9–22; Figure 7). However, the analysis of clinical data taking into account the most relevant prognostic features in this advanced setting (i.e., residual disease and histotype) indicated that USP8 is an independent prognostic factor for adverse outcome when considering PFS as clinical end-point (HR:1.86, 95%CI:1.03–3.33, *p* = 0.038; Table 3 for univariate and multivariate Cox regression analysis). IHC staining of normal ovarian cells (i.e., Fallopian tube secretory cells) indicated the expression of USP8 in normal cells (Supplementary Figure S5).

**FIGURE 7:**
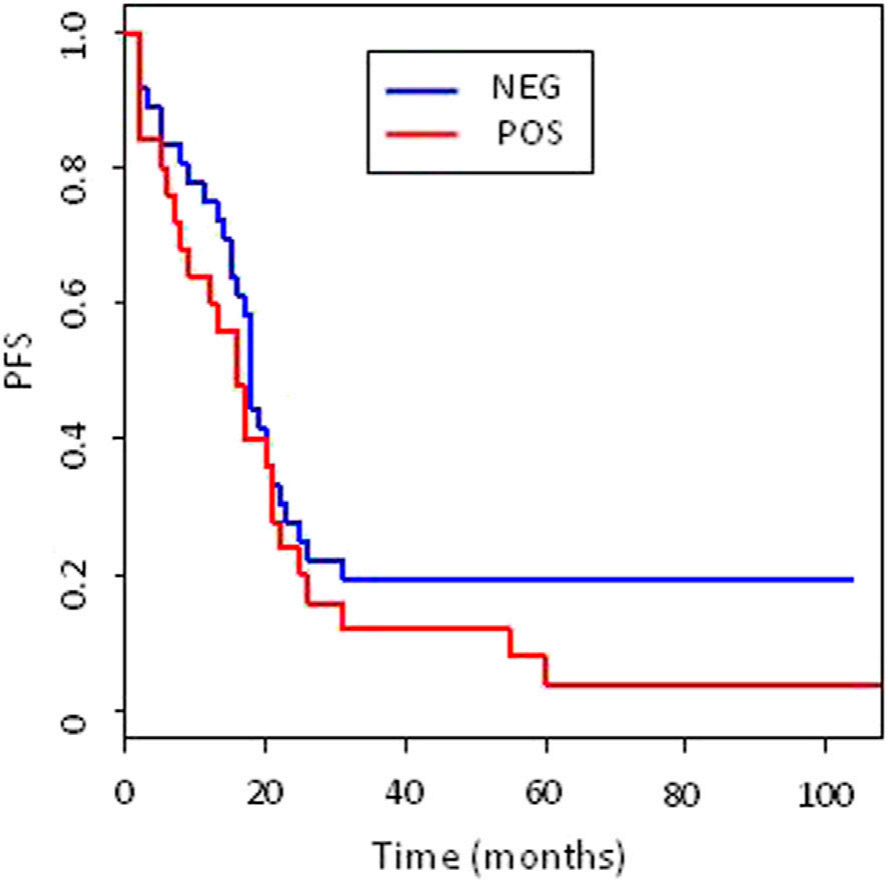
Kaplan Meier (KM) curves of Progression free survival (PFS). Patients were stratified according to USP8 expression (blue lines negative, red line positive). KM curves compared with log-rank *p* test resulted not significantly different. Number of events and median PFS (with 95%CI) are reported for each group.

**TABLE 3:** Univariate and multivariate analysis (Cox regression) of PFS for clinical and biological (USP8) variables.^a^

	*Univariate analysis*			*Multivariate analysis*		
	HR	(95%CI)	p*	HR	(95%CI)	p*
*Surgical debulking*						
SOD vs. OD	2.66	1.48–4.79	*0.001*	3.5	1.76–6.95	*0.0003*
*Histotype*						
HGSOC vs. others	1.36	0.76–2.42	*0.29*	0.73	0.38–1.41	*0.35*
*USP8 expression*						
Pos vs. Neg	1.35	0.79–2.33	*0.272*	1.86	1.03–3.33	*0.038*

^a^ SOD, suboptimal debulking; OD, optimal debulking; HGSOC, high grade serous ovarian carcinomas; HR, hazard ratio; CI, confidence interval; *p*, *p* value from statistical anlyses.

## Discussion

In spite of the initial response to platinum-based chemotherapy, ovarian cancer often acquires drug resistance (Romero and Bast, 2012; Bowtell et al., 2015; Herzog and Monk, 2017). This phenomenon is considered to involve multiple players among which DUBs are emerging as important regulators (Clague et al., 2019). Indeed, DUBs can interplay with survival pathways, which are hyper-activated in platinum-resistant cells (Cossa et al., 2014; Corno et al., 2017). In this context, USP8 could play a role by modulating RTK signaling. In fact, poor termination of receptor signaling may lead to enhanced signaling. In addition, USP8 has been implicated in regulation of apoptosis (Panner et al., 2010; Jeong et al., 2017).

At present, it is still poorly understood if USP8 can contribute to survival of cancer cells independently of their drug sensitivity. In fact, CRISPR–Cas9 essentiality screens in cancer cells (Meyers et al., 2017) suggest that USP8 has a general relevance in cell viability as also evidenced by its physiological role, which appears non-redundant. Indeed, embryonic lethality has been observed in USP8 knockout mice (Dufner and Knobeloch, 2019). In a recent study, we observed a variable expression of USP8 across ovarian cancer cell lines and found a synergistic interaction between cisplatin and caffeic acid phenethyl ester, identified as USP8 inhibitor, in endometrioid ovarian carcinoma cells expressing high levels of USP8, thereby suggesting the opportunity to carry out a molecular targeting of USP8 in such a disease (Colombo et al., 2022). Thus, the present study was designed to examine the contribution of USP8 to drug resistance and response to cisplatin given the possible interplay of USP8 with survival pathways.

When we examined USP8 expression levels and activity in different ovarian carcinoma cell lines including pairs of cisplatin sensitive and resistant cells, we found increased levels in the latter and enhanced activity in cells representative of the endometrioid ovarian carcinoma subtype. Sequencing of IGROV-1, IGROV-1/Pt1, A2780, A2780/CP and A2780/BBR cDNA fragments (data not shown) indicated that increased activity was not due to gene mutations that have been described to provide increased USP8 catalytic activity in Cushing’s disease resulting in activation of EGF-R signaling (Reincke et al., 2015), but likely reflected increased USP8 protein levels. Enhanced expression/activity of USP8 was not a general feature of cisplatin-resistant cells, because it was not found in the cisplatin-resistant PEO4 and PEO6 variants, in which resistance is associated to BRCA2 reversion mutation. When considering all the examined cell lines, overall no direct relation between mRNA and protein level was found. It can be speculated that a repression of USP8 proteasome-mediated degradation may occur in resistant cells displaying increased protein levels and activity (e.g., IGROV-1/Pt1 and A2780/BBR), whereas a stimulation of protein degradation may occur in the PEO4 and PEO6 variants. Of note, an analysis of the levels of genes underlying pluripotency/stemness/EMT indicated that enrichment of USP8 was associated with increased levels of selected genes of these classes only in the A2780 variants, suggesting the lack of a general link between USP8 and stemness. Our findings are also in keeping with the multifactorial nature of the drug resistance mechanisms that are known not to be homogeneous across cellular models and patients (Rottenberg et al., 2021).

An analysis of survival-related features of the cisplatin-resistant IGROV-1/Pt1 cells upon USP8 molecular targeting, indicated that USP8 interplays with RTKs since its silencing resulted in reduced activation of all RTKs belonging to the Human Epidermal Growth Factor Receptor (HER) family i.e., ErbB1/EGF-R, ErbB2, ErbB3 and ErbB4, and as a consequence decreased Akt activation as shown by reduced phosphorylation at Ser473. The interplay between USP8 and Akt has been poorly studied and few and conflicting data have been reported. In fact, Akt activation has been shown to decrease USP8 function in glioblastoma cells (Panner et al., 2010), whereas in breast tumor cells Akt activation appears to lead to increased USP8 activity (Cao et al., 2007). Our findings are in keeping with the latter report and the view that reduced USP8 level may decrease activation of RTKs and as a consequence of the downstream PI3K/Akt pathway.

Molecular targeting of USP8 revealed a link between FLIP_L_ and USP8, with down-regulation of FLIP_L_ upon USP8 silencing resulting in enhanced susceptibility to cisplatin-induced apoptosis as observed by Annexin V-binding assays in USP8-silenced cells. Increased apoptosis appeared to be a consequence of enhanced activation of caspases 3/7 and to a lesser extent of caspase 8 whose activation was significantly increased only with siRNA b that gave the higher USP8 targeting efficacy. The pattern of caspase activation implies a major contribution of the intrinsic pathway to cell death, but does not exclude the extrinsic pathway that may be engaged before the intrinsic one. Modulation of FLIP_L_ levels is in keeping with the available evidence indicating that USP8 counteracts death receptor-mediated apoptosis by increasing FLIP_L_ stability. Indeed, direct deubiquitination of FLIP_L_ has been shown to suppress proteasome-dependent degradation (Panner et al., 2010). Our results are also consistent with the finding that USP8 inhibits extrinsic apoptosis because when using the most efficient siRNA i.e., siRNA b to inhibit USP8, cisplatin-induced caspase 8 activation was evident.

Molecular targeting of USP8 was also carried out in the IGROV-1 parental cell line and in the PEO1 cells. In both cell lines USP8 silencing resulted in increased susceptibility to cisplatin-induced apoptosis in keeping with the hypothesized general role of this DUB in tumor cell survival independently of the relative resistance to cisplatin. In this regard, although an indicative assessment of apoptosis activation is obtained by the western blotting, a quantitative measurement is provided by the Annexin V-binding assay. A contribution of USP8 to physiological cell viability can also be evinced from the basal levels of apoptosis observed in USP8-silenced PEO1 cells, which were higher than those of untransfected and negative control transfected cells. Such a difference was not evident in IGROV-1 and IGROV-1/Pt1 cells, likely depending on the different molecular background.

Overall, the mechanism by which USP8 reduces cisplatin activity is linked to a change in tumor cell susceptibility to apoptosis. This behavior involves 1) the down-regulation of survival signal triggered by RTKs and of anti-apoptotic proteins such as FLIP_L_ and Bcl-2 in untreated IGROV-1/Pt1 cells, 2) a decrease of the anti-apoptotic claspin and survivin in IGROV-1 cells treated with cisplatin, 3) an enhanced basal activation of caspase three in PEO cells. Of note, IGROV-1/Pt1 silenced cells tried to activate a protective response upon cisplatin exposure (particularly with 3 µM cisplatin) by increasing Bcl-2. Thus, the modulated factors appear to be dependent on the molecular background of the cell lines.

To define the possible clinical relevance of our preclinical results, we also carried out an exploratory IHC analysis of USP8 in ovarian carcinoma clinical samples. With this approach, we identified that a large subset of ovarian carcinomas were USP8 positive. This finding might appear unexpected given the physiological role of USP8 (Dufner and Knobeloch, 2019). However, it is conceivable that some tumors may lose or down-regulate USP8 during progression. When exploring the clinical role of USP8, we found that it was an independent prognostic factor for adverse outcome, in keeping with reports on cervical squamous cell carcinoma in which high expression is related to poor survival (Yan et al., 2018). Although our preclinical results show that USP8 finely tunes cisplatin response, it has been reported that even small changes of sensitivity *in vitro* (e.g, see IGROV-1/Pt1 colony forming assays) can translate into clinically relevant changes (Fink et al., 1996; Lin et al., 2001). In addition, the espression of USP8 in Fallopian tube secretory cells highlights a physiological role of USP8 in maintaining cell viability in keeping with reports from the literature as mentioned above (Meyers et al., 2017; Dufner and Knobeloch, 2019). However, since USP8 acts on substrates that might preferentially expressed in tumors, USP8 role may in part differ between normal and tumor cells.

Our findings show that molecular targeting of USP8 in ovarian carcinoma cells results in improved apoptotic response to cisplatin. Of note, this effect is achieved both in platinum sensitive and –resistant cells in cell lines representative of two important histological subtypes, the endometrioid (IGROV-1 and IGROV-1/Pt1 cell lines) and high grade serous (PEO1). Although the different co-players and targets of USP8 may in part vary in different cell lines, the preclinical results reported here, together with our translation study in clinical specimens is in keeping with the recent literature showing a role for USP8 in cancer progression and increased USP8 in patients resistant to neoadjuvant therapies (Xie et al., 2022) and highlight promising features of USP8 as a possible ovarian carcinoma biomarker.

## Data Availability

The raw data supporting the conclusions of this article will be made available by the authors, without undue reservation.

## Abbreviations

EGFR, Epidermal Growth Factor Receptor; RTKs, Receptor Tyrosine Kinases; DUBs, Deubiquitinating enzymes; USP, Ubiquitin-Specific Protease; Ub, ubiquitin; IHC, Immunohistochemistry; FFPE, Formalin-Fixed Paraffin-embedded; EOC, Epithelial Ovarian Carcinoma; PFS, Progression Free Survival; HR, Hazard Ratio; OD, Optimal Debulking; SOD, Suboptimal Debulking; EMT, Epithelial-Mesenchimal Transition.
