# Supplementary material for: The deubiquitinase USP8 regulates ovarian cancer cell response to cisplatin by suppressing apoptosis
Source: Front Cell Dev Biol. 2022 Dec 12;10:1055067. doi: 10.3389/fcell.2022.1055067 (PMC9791127; doi:10.3389/fcell.2022.1055067)
Supplement: Supplementary file 1 [file Table1.DOCX]

Supplementary Material

**1 Supplementary Figures and Tables**

- 1. **Supplementary Table**

**Supplementary Table 1. Quantification of dot intensity of Human Phospho-RTKs array in IGROV-1/Pt1 cells after USP8 molecular targeting.**

| Sample | p- EGFR | p- ErbB2 | p- ErbB3 |
| --- | --- | --- | --- |
| Untransfected | 2816.569 | 2442.154 | 12987.711 |
| Negative Control | 3659.397 | 4724.518 | 14613.021 |
| USP8 siRNA b | 2265.861 | 1276.790 | 10782.347 |
| USP8 siRNA c | 4123.983 | 4335.447 | 13561.953 |

Dot intensity quantification was performed using ImageJ**.**

| Sample | survivin | claspin | p- p53 (S46) |
| --- | --- | --- | --- |
| Negative Control  Untreated | 9993.702 | 10176.146 | 2571.589 |
| Negative Control  1µM cisplatin | 6600.660 | 8860.933 | 8660.782 |
| USP8 siRNA  Untreated | 10114.903 | 10523.832 | 2994.811 |
| USP8 siRNA  1µM cisplatin | 6071.69 | 7857.347 | 8567.811 |

**Supplementary Table 2. Quantification of dot intensity of Human Apoptosis array in IGROV-1 cells after USP8 molecular targeting.**

Dot intensity quantification was performed using ImageJ**.**

## Supplementary Figures


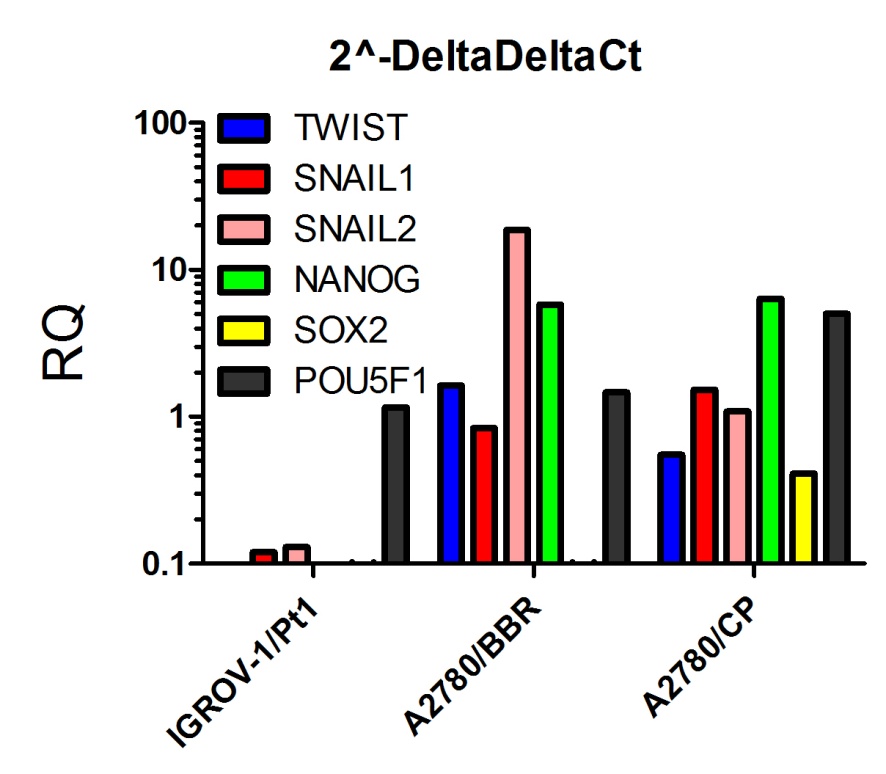


**Supplementary Figure 1. Analysis of mRNA levels of pluripotency/stemness and epithelial mesenchymal transition genes**. The analysis was carried out by qRT-PCR. Relative quantification is shown on the y axis. Each parental cell line was used as calibrator. GAPDH was employed as housekeeping gene.


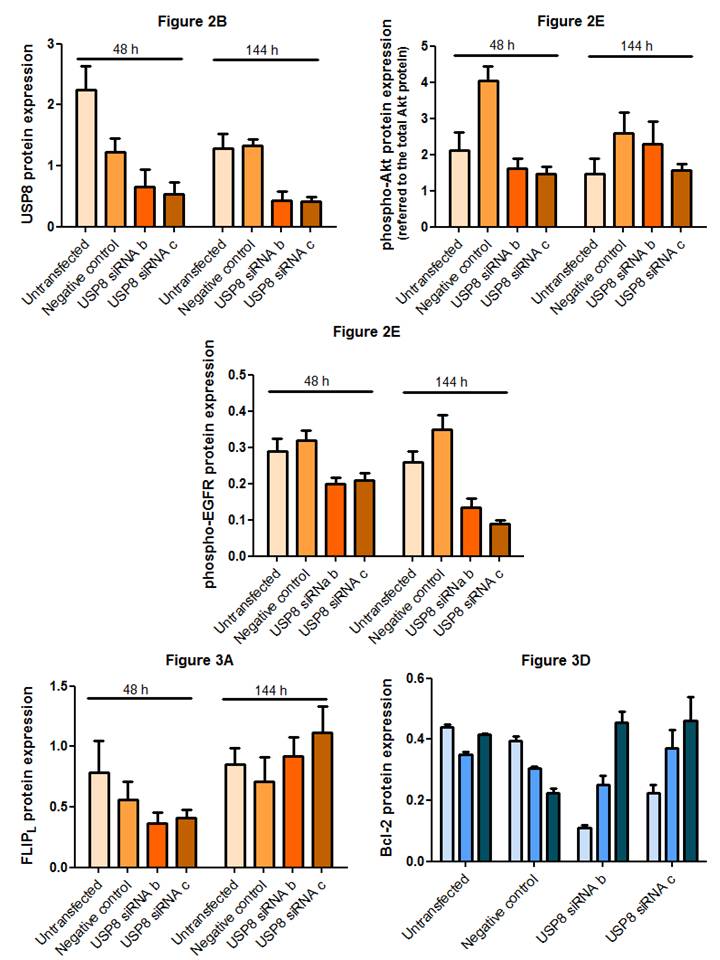


**Supplementary Figure 2.** **Analysis of protein levels in IGROV-1/Pt1 cells after USP8 molecular targeting.** Band intensity of western blot analysis, shown in the manuscript (Figures 2-3), was quantified using ImageJ and the obtained mean values (mean ± SEM, n=3) are reported in the histograms. Statistical analysis provides the following *P* values by unpaired Student’s t test: USP8 - *P* = 0.08 (negative control 48 h *versus* siRNA c 48 h), *P* < 0.01 (negative control 144 h *versus* siRNAs 144 h); phospho-EGFR – *P* < 0.01 (negative control 48 h *versus* siRNA b 48 h), *P* < 0.05 (negative control 48 h *versus* siRNA c 48 h and negative control 144 h *versus* siRNAs 144 h); phospho-Akt - *P* < 0.01 (negative control 48 h *versus* siRNAs 48 h); Bcl-2 – *P* < 0.05 (negative control untreated cells *versus* siRNAs untreated cells).


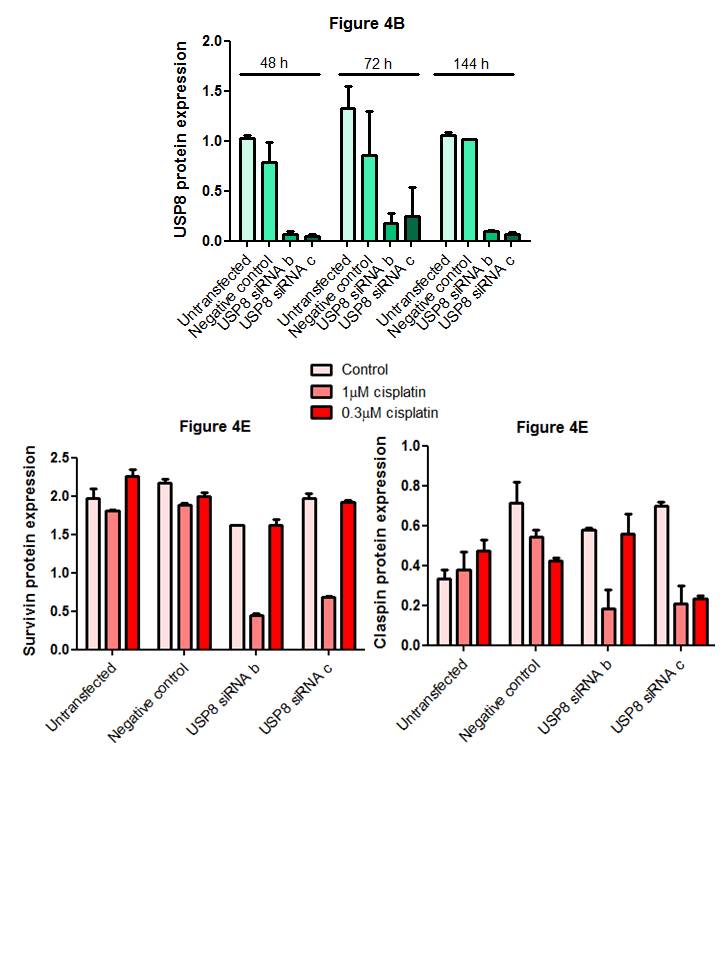


**Supplementary Figure 3.** **Analysis of protein levels in IGROV-1 cells after USP8 molecular targeting.** Band intensity of western blot analysis, shown in the manuscript (Figure 4), was quantified using ImageJ and the obtained mean values (mean ± SEM, n=3) are reported in the histograms. Statistical analysis provides the following *P* values by One-Way ANOVA followed by Bonferroni’s test for multiple comparisons: USP8 - *P* < 0.0001 (negative control 48-144 h *versus* siRNAs 48-144 h respectively), *P* = 0.05 (negative control 72 h *versus* siRNAs 72 h); survivin - *P* < 0.0001 (negative control cells treated with 1µM cisplatin *versus* siRNAs cells treated with 1µM cisplatin); claspin – *P* = 0.05 (negative control cells treated with 1µM cisplatin *versus* siRNAs cells treated with 1µM cisplatin).

**
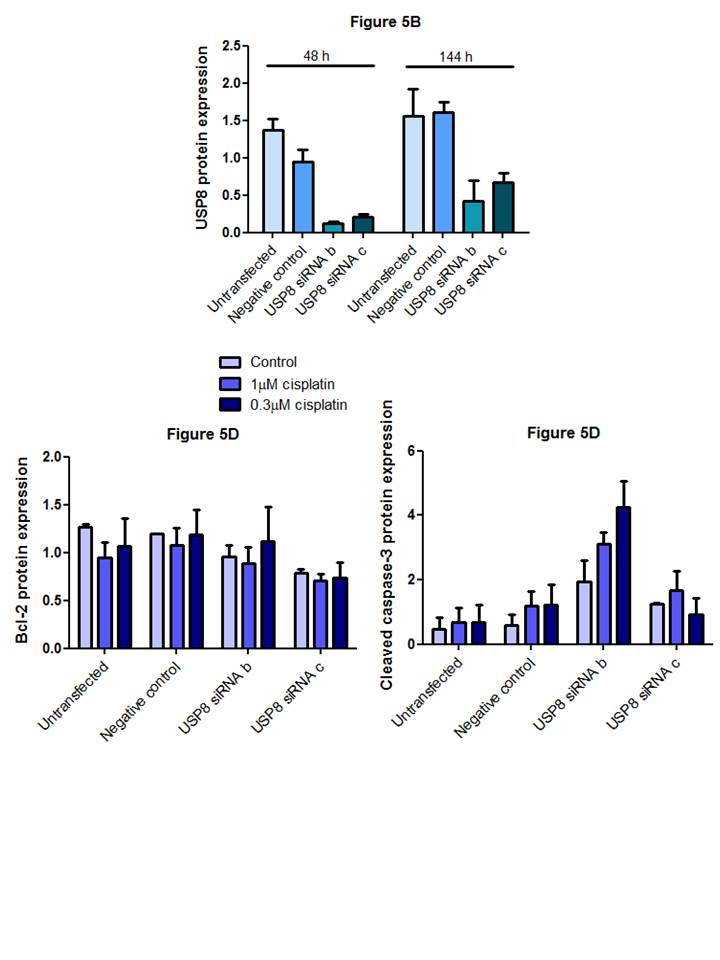
**

**Supplementary Figure 4.** **Analysis of protein levels in PEO1 cells after USP8 molecular targeting.** Band intensity of western blot analysis, shown in the manuscript (Figure 5), was quantified using ImageJ and the obtained mean values (mean ± SEM, n=3) are reported in the histograms. Statistical analysis provides the following *P* values by unpaired Student’s t test: USP8 - *P* = 0.08 (negative control 48 h *versus* siRNA c 48 h), *P* < 0.01 (negative control 144 h *versus* siRNAs 144 h ); Bcl-2 - *P* < 0.0001 (negative control cells treated with 1µM cisplatin *versus* siRNAs cells treated with 1µM cisplatin).

**Supplementary Figure 5. Immunohistochemical evaluation of USP8 in a fallopian tubes specimen.** Representative image of a fallopian tube sample, showing that the protein of interest is expressed in normal cells like Fallopian tube secretory cells. Negative control, slides incubated with secondary antibody alone.
